# Clinical and economic value of sofosbuvir-based regimens in the treatment of chronic hepatitis C in Spain

**DOI:** 10.1371/journal.pone.0278544

**Published:** 2022-12-01

**Authors:** Rafael Esteban, Raquel Domínguez-Hernández, Victoria Martín-Escudero, Miguel Ángel Casado

**Affiliations:** 1 Liver Unit, Hospital Universitari Vall d’Hebron, Barcelona, Spain; 2 CIBERehd, Instituto Carlos III, Barcelona, Spain; 3 Pharmacoeconomics & Outcomes Research Iberia (PORIB), Madrid, Spain; 4 Gilead Sciences, Madrid, Spain; Istituto Superiore di Sanità and St. Camillus International University of Health Sciences, ITALY

## Abstract

**Background:**

The treatment of chronic hepatitis C virus (HCV) with direct-acting antivirals has undergone a spectacular revolution and added significant value to healthcare systems and patients. The aim of the study was to evaluate the efficiency and value of Sofosbuvir (SOF)-based regimens for a target population of 85,959 chronic HCV patients treated in Spain during 2015–2019, compared to previous therapeutic strategies (peginterferon/ and ribavirin in double/triple therapy with telaprevir or boceprevir).

**Methods:**

A previously developed lifetime Markov model was adapted to simulate the disease HCV evolution. In SOF-based regimens, all patients (100%) were treated regardless with sustained virological response (SVR) of 93–98%, obtained from real-world data. In previous therapeutic, only ≥F2 patients were treated according to clinical practice (38%) with an average SVR of 61% taken from published literature. The value was measured as clinical and economic impact in terms of avoided HCV-related mortality and liver complications; total costs and quality-adjusted life years (QALYs) applying an annual 3% discount rate.

**Results:**

Compared to previous therapeutic, during lifetime, SOF-based regimens reduced decompensated cirrhosis by 89%, hepatocellular carcinoma by 77% and liver transplant by 84%, decreasing the cost associated to liver complications management in €770 million. SOF-based regimens also decreased liver-related mortality by 82%. Besides, SOF-based regimens gained 310,765/QALYs, saving €274 million (considering drugs, monitoring, and HCV management).

**Conclusion:**

For Spain, SOF-based regimens offer value for HCV patients in terms of lowering HCV-related liver disease burden and generating significant cost savings for the health system, contributing to the WHO goal.

## Introduction

Approximately 58 million people worldwide have chronic hepatitis C virus (HCV) infection [WHO]. In its chronic phase, the disease can cause serious long-term liver complications such as cirrhosis, liver cancer, and even the need for a liver transplant [1], being one of the most common causes of liver mortality and morbidity [2, 3]. In addition, it represents a significant economic burden on health systems, associated not only with the cost of complications and liver transplantation [4, 5] but also with the high comorbidity, the use of concomitant medication in young patients (> 45 years) [6] and extrahepatic manifestations [7–9].

The availability direct-acting antivirals (DAAs) since 2015 has changed the paradigm of chronic hepatitis C (CHC) treatment. Sofosbuvir was the first DAA used in the therapeutic arsenal. With previous therapies based on pegylated interferon (PEG-IFN) in combination with double or triple therapy with telaprevir or boceprevir, survival virological response (SVR) rates were low, depending on the genotype, and not all patients had access to treatment or required long follow-up [10]. The new DAAs based on sofosbuvir provide important clinical benefits, with a high effectiveness (SVR12> 95%), which leads to a reduction in mortality, an improvement in liver function, a delay in the appearance or the disappearance of cirrhotic complications (ascites, hypertension reduction) and even prevention of liver transplantation in some cases [11–13]. They have a shorter duration of administration than previous regimens, with good tolerance, and cause fewer adverse effects [14]. In addition, they have allowed the early treatment of the disease, since they can be given from mild initial stages of fibrosis (F0-F4), even more, the sofosbuvir/velpatasvir with or without voxilaprevir regimens are pangenotypic, and the simplification in their administration has facilitated access to treatment for more patients [15]. Likewise, the use of DAAs has led to a decrease in the prevalence of HCV viremia in recent years [16], although there are still certain population groups, such as injected drug users, with high prevalence. The use of DAAs, in conjunction with other prevention and detection interventions, has produced a reduction in HCV transmission [17].

The emergence of DAAs also motivated the WHO to set the goal of eliminating hepatitis C, proposing to reduce new infections by 90% and mortality by 65% by 2030 [18]. Given the public health problem posed by hepatitis C, in Spain in 2015 the Strategic Plan for the Approach to Hepatitis C (PEAHC) was established. Since then, the creation by various organs not only of clinical guidelines for the assessment and use of new therapies but also of strategies and recommendations that promote the diagnosis of the disease [15, 19, 20] has made it possible to detect hidden infections and treat them. Therefore, based on the evolution of treatment so far and based on the benefits of DAAs, Spain could be one of the first countries to achieve the elimination of hepatitis C [21].

The objective of this analysis was to evaluate the clinical and economic value of sofosbuvir-based regimens (SOF-regimens) as therapy for CHC during the period between 2015 and 2019 compared to previous therapeutic strategies in Spain.

## Methods

An economic evaluation was performed through modelling to evaluate the efficiency of sofosbuvir-based regimens (SOF-regimens) in patients with CHC, comparing two treatment alternatives: SOF-regimens (Harvoni^®^, Epclusa^®^, Vosevi^®^, and Sovaldi^®^ in combination with daclatasvir or simeprevir) compared to previous therapeutic strategies (PEG-IFN and ribavirin in double or triple therapy with telaprevir or boceprevir). The target population, 85,959 patients, was obtained from the total population of chronic patients treated with SOF-based therapies during 2015–2019 in Spain [22, 23].

For the analysis, we adapted a previously validated and published Markov model that simulated the annual progression of chronic patients through the different health states of the disease (mild, moderate and advanced fibrosis; SVR; decompensated cirrhosis; hepatocellular carcinoma; liver transplant; and posttransplant) until death [4]. To perform the simulation, the same parameters on transition probabilities, utility values and costs of each health state were used as those included in the published model (S1 Table in S1 File) [4]. The values of mortality by all causes and by hepatic causes were updated according to the latest data published in Spain [24].

Patients, who had a mean age of 45 years [22], were incorporated into the model annually (35% for 2015, 23% for 2016, 19% for 2017, 13% for 2018 and 10% for 2019), distributed by fibrosis states from F0 to F4. This distribution varied annually according to the year of treatment [22] (Table 1), with the percentage of cirrhotic patients ranging from 14–50% [22]. With SOF regimens, 100% of patients are treated regardless of their fibrosis status (F0-F4). The SVR rates of these regimens, obtained from real-world studies, varied between 93.8 and 98.5% [25–30]. With the previous therapeutic strategies, following clinical guidelines, only patients in states ≥F2 have access to treatment, representing 38% of the total target population (9,800 per year) [31]. The average SVRs for these strategies were derived from published studies (60.6–61.2%) [4, 5] (Table 1). Untreated patients progress in the simulation according to the natural history of the disease.

**Table 1:** Analysis parameters.

Parameter	Value	Reference
Distribution by fibrosis states	Minimum and maximum value range (2015–2019)	
F0	0.71–4.23%	[22]
F1	7.02–62.58%	[22]
F2	13.49–33.39%	[22]
F3	8.64–20.36%	[22]
F4	11.53–50.47%	[22]
**Therapies previusly SVR**		
F2	61.2%	[4]
F3	61.2%	[4]
F4	60.6%	[4]
**SOF- Regimen SVR**		
Sovaldi^®^	93.8%	[25–30]
Harvoni^®^	95.8%	[25–30]
Epclusa^®^	98.5%	[25–30]
Vosevi^®^	95.0%	[25–30]

SOF, sofosbuvir; SVR, survival virological response

The direct health costs associated with each strategy included the average pharmacological cost (€16,023, SOF-regimens obtained from real-world data; €15,003, previous therapeutic strategies [4], the cost of treatment monitoring (€264, SOF-regimens [32]; between €2,371 and €2,466€, previous therapeutic strategies [4] and the costs of disease management, common by both strategies [4]. The total cost was the sum of the three costs. All costs were updated to 2020, according to the interannual variation of the published Consumer Price Index [33].

The value of the SOF-regimens was evaluated based on the comparison of both strategies from the perspective of the National Health System (NHS). The results are shown for a time horizon of the entire life of the patients in clinical terms (mortality and hepatic complications avoided), economic (costs associated with hepatic complications) and efficiency (total costs and quality-adjusted life-years, QALYs). An annual discount rate of 3% was applied to all results [34].

In addition, a monetary estimate was made of the social value of the QALY gain [35] obtained from the most effective alternative compared to the least effective. The calculations were performed by multiplying the total number of incremental QALYs by the willingness to pay, taking into account different efficiency thresholds: 20,000, 25,000 and 30,000 euros per QALY [36, 37].

Additionally, to evaluate the robustness of the results of the analysis, univariate and multivariate deterministic sensitivity analyses (DSA) were performed by modifying the most significant parameters. The univariate DSA included: the SVR rate for DAAs (95%) (DSA1) [22] and for previously therapies (55.0–75.2%) (DSA2) [4–5], DAAs costs (±20%) (DSA3), previously therapies (±20%) (DSA4) the cost of treatment monitoring (± 20%) (DSA5) and the variation of the discount rate (0% and 5%) [36] (DSA6). Multivariate DSA parameters were [4–5], utility values (DSA7) [4–5] and health costs associated with each health (±20%) (DSA8).

## Results

**Fig 1:**
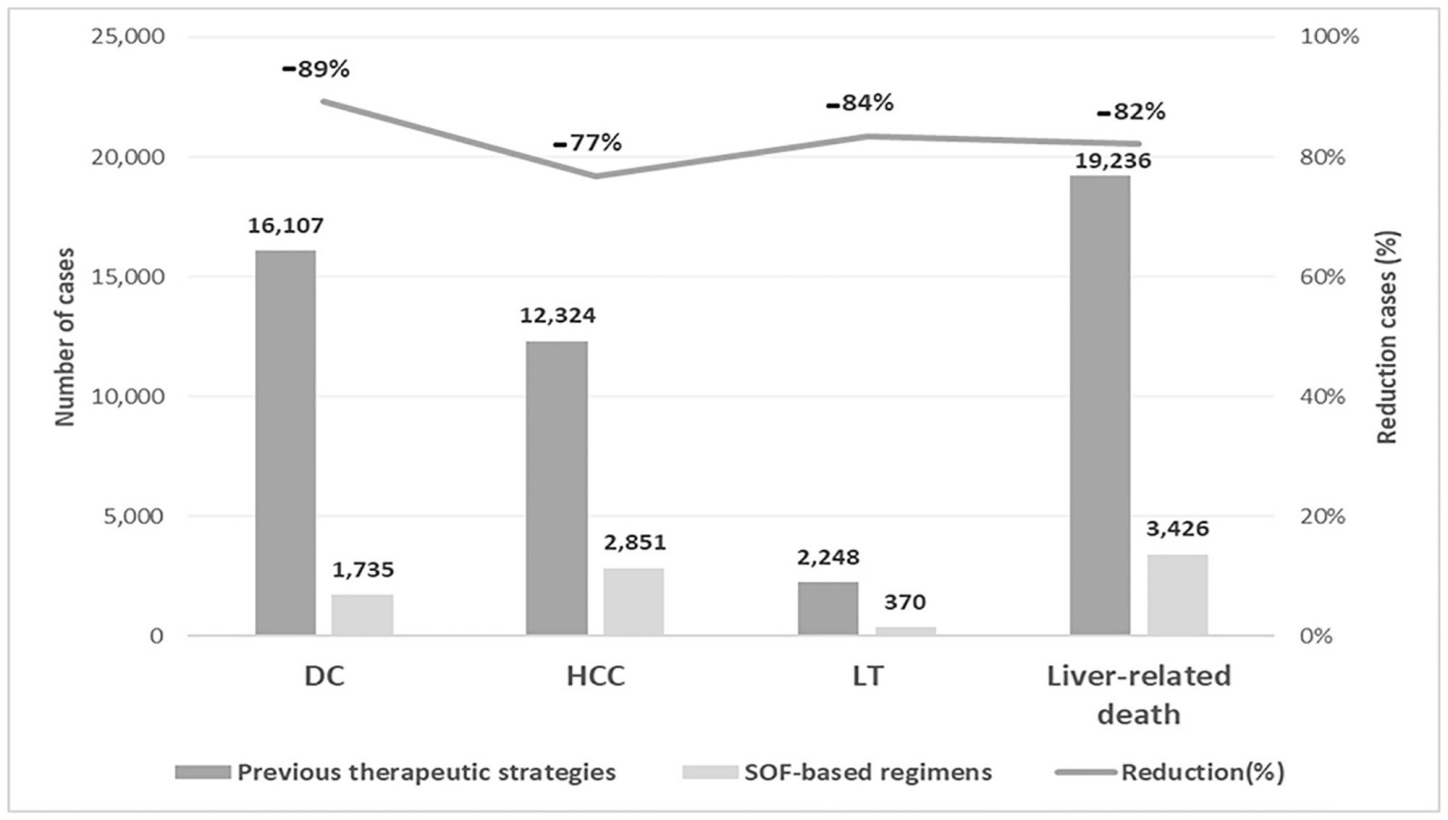
Number of clinical events and reduction in clinical events cases between both strategies for the total cohort. DC, decompensate cirrhosis; HCC, hepatocellular carcinoma; LT, liver transplant.

**Fig 2:**
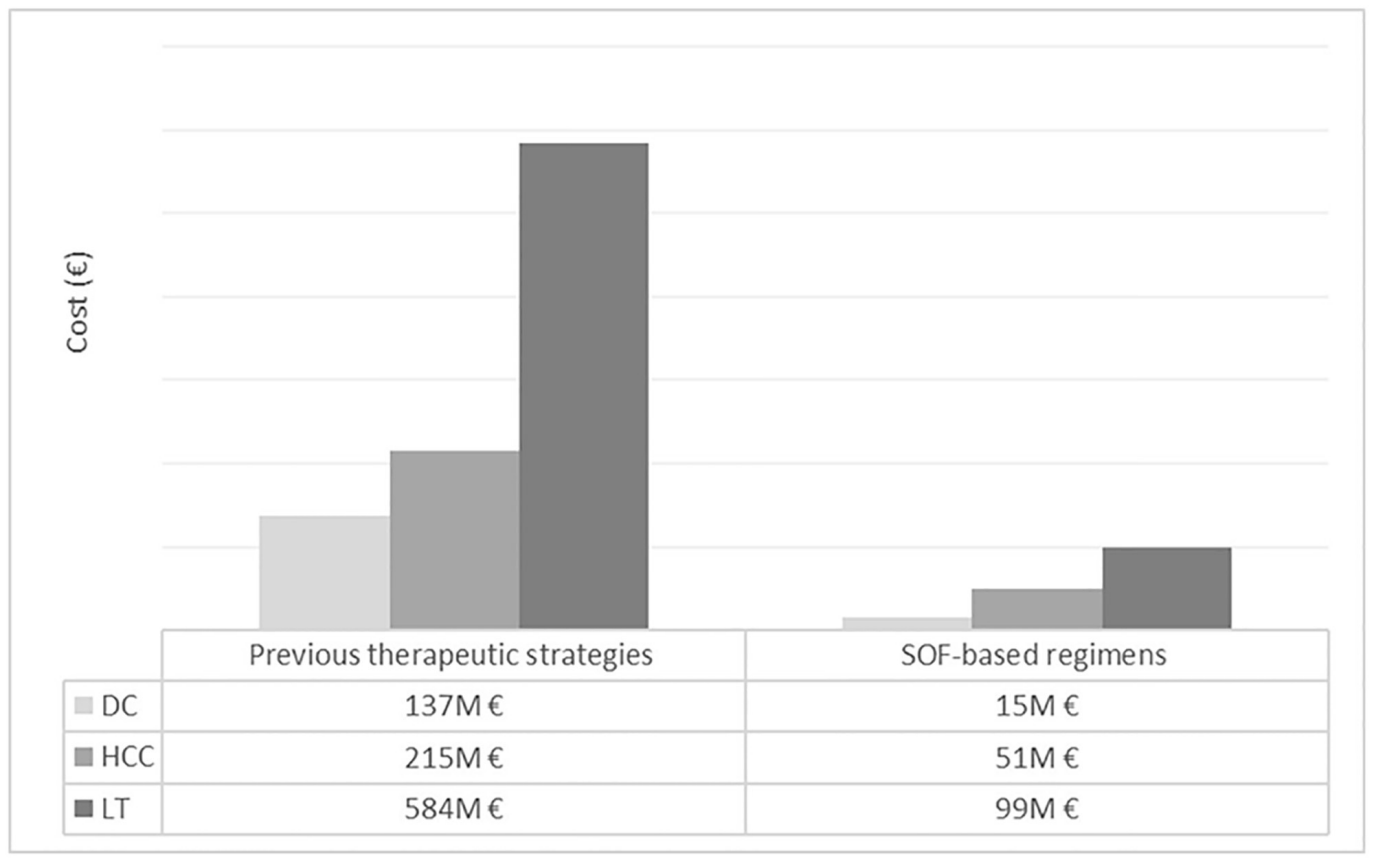
Liver-related complications cost for the total cohort. DC, decompensate cirrhosis; HCC, hepatocellular carcinoma; LT, liver transplant.

Compared with the previous therapeutic strategies, during the entire time horizon of the analysis, SOF-regimens decreased hepatic mortality by 82% (-15,810 cases). Cases of decompensated cirrhosis were reduced by 89% (-14,372), cases of hepatocellular carcinoma by 77% (-9,473) and patients who would need a liver transplant by 84% (-1,878) (Fig 1). In economic terms, there was a cost savings associated with these hepatic complications of € 770 million with the use of SOF-regimens compared to previous therapeutic strategies (Fig 2).

**Fig 3:**
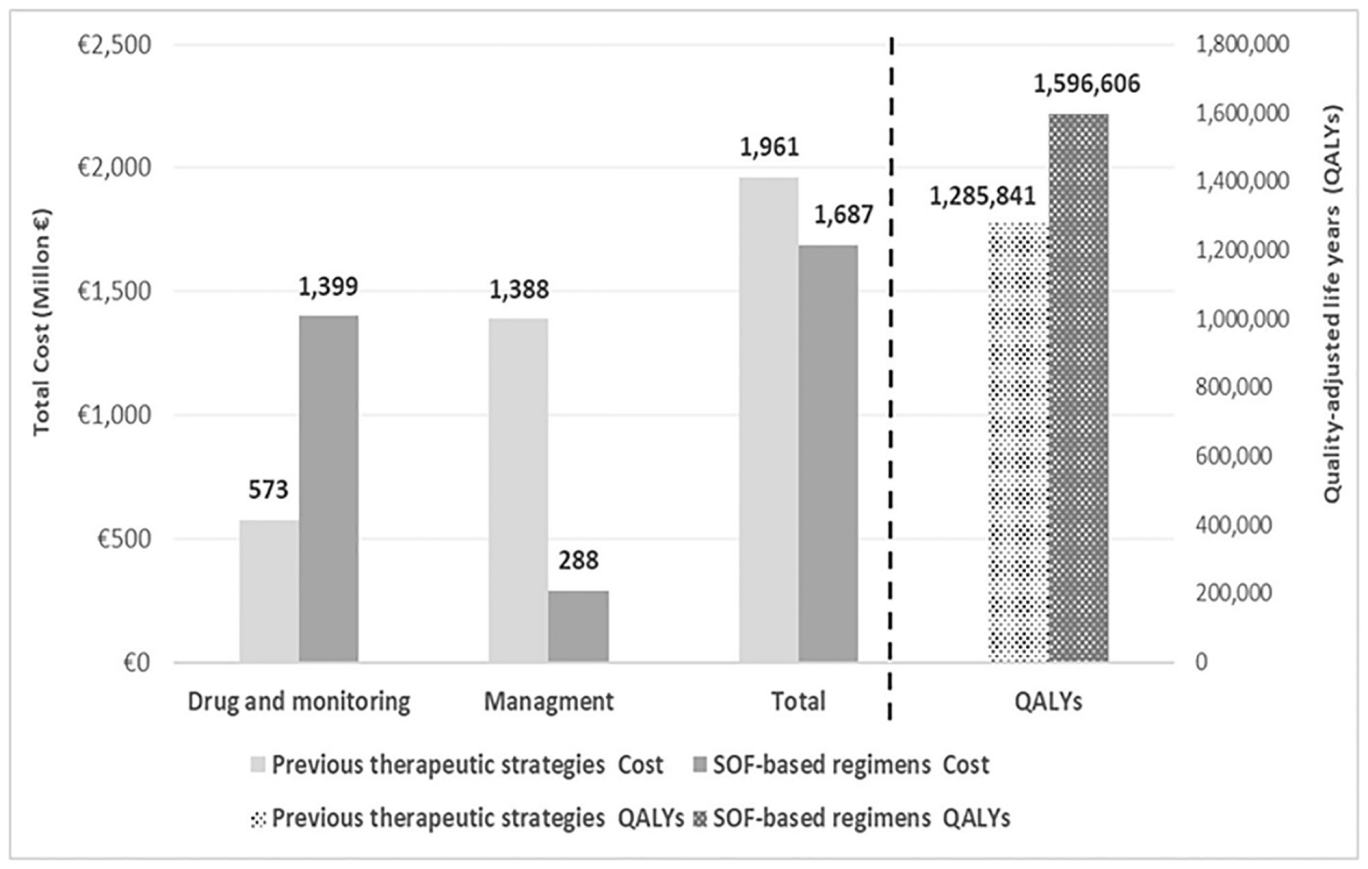
Cost-effectiveness results for the total cohort. SOF, sofosbuvir; QALYs, Quality-adjusted life years.

The results of the cost-effectiveness analysis in which the SOF-regimens were compared with the previous therapeutic strategies showed an increase in the entire cohort of 310,765 QALYs and a savings of 274 million euros (Fig 3).

The monetary value of the increase in QALYs achieved with the SOF-regimens in the total population of patients treated compared to previous therapeutic strategies, using the number of incremental QALYs and considering an availability to pay between €20,000 and €30,000 per QALY gained, ranged between 6,215 and 9,323 million euros. With the SOF-regimens, a return on investment of between €4.5 and €6.8 would be obtained for each euro invested, considering a cost per QALY gained between € 20,000–30,000.

The sensitivity analyses showed that the results described above were robust. The QALYs values ranged between 306,207 and 964,856 additional QALYs, and the incremental cost ranged between 175 and 1,272 million euros. The parameter with the greatest influence on the QALYs results were utility values and SVR rates of both treatments. The parameter with the greatest influence on the results of total costs were drug and management costs. The discount rate was the parameter with the greatest impact on both clinical and economical results. The variation in QALYs and total costs when each parameter is changed with the maximum and minimum values were shown in S2 Table in S1 File.

## Discussion

Hepatitis C is a global public health problem with a significant clinical burden associated with its morbidity and mortality, which generates high costs to the health system. The advent of DAAs, including SOF-regimens, and the involvement of different health agents to achieve the objectives of the WHO have allowed the treatment and cure of a large number of patients with CHC. Therefore, our analysis evaluated the clinical and economic benefits of patients treated with SOF-regimens between the years 2015 and 2019 in Spain.

The analysis shows that treatment with SOF-regimens achieves significant clinical and economic benefits. The obtaining of such high SVR rates compared to previous therapeutic strategies is reflected in the decrease in cases of liver decompensation, hepatocellular carcinoma and indications for liver transplants, in addition to liver mortality. Likewise, this decrease in the clinical burden associated with liver complications leads to a reduction in the economic burden, producing substantial savings for the NHS. In addition, the use of SOF-regimens compared to previous therapeutic strategies is cost-effective, resulting in greater effectiveness measured in QALYs and a reduction in total costs.

The analysis shows the efficiency of the SOF-regimens in a real cohort of patients already treated. Together with the clinical characteristics and response rates obtained from clinical practice and evaluated by official bodies, our findings give robustness to the results in the form of an actual estimate. In addition, these results highlight the effort made by health organizations and professionals to treat and cure the greatest number of patients with CHC.

Other studies that have analysed the efficiency of DAAs have shown similar results of health outcomes, showing that they are greater than those yielded by previous therapies and that DAA treatment would reduce the costs of the disease, generating savings in the cost of health care and improving long-term health [4, 5, 38, 39].

The present analysis has some limitations. One of them derives from the inclusion of direct health costs exclusively, without considering the evaluation of the impact of the use of SOF-regimens on productivity and absence from work, compared to previous therapeutic strategies. Other studies evaluating the indirect costs associated with DAAs have shown that their use generates an increase in productivity and less absence from work [40, 41]. In our analysis, these costs were not included, but they are of great importance in the evaluation of the disease as a whole, and their inclusion would have generated a greater benefit. Subsequent studies should evaluate the indirect costs linked to increased productivity and reduced absence from work, in addition to direct cost savings. Another limitation is that the comorbidity associated with hepatitis C generates a series of extrahepatic manifestations not considered in this analysis. There are studies that show that the use of DAAs reduces the risk of suffering hepatitis C comorbidities and their costs [7, 8]. Their inclusion would have benefited the strategy based on the SOF-regimens since these regimens have a lower comorbidity burden than previous therapies, which would have decreased their cost.

On the other hand, it should be noted that the use of DAAs has contributed to the elimination of hepatitis C in Spain. The diversity of measures implemented to favour the detection and diagnosis of hidden infections, such as point-of-care and one-step diagnosis, among others, have made it possible to increase screening and access to treatment.

## Conclusion

Treatment with regimens based on SOF has achieved a significant reduction in long-term clinical events and mortality due to HCV, which contributes to the objectives of the WHO of eliminating hepatitis C. In addition, treatment with SOF- regimens reduces the economic burden of the disease and generates significant savings for the NHS.

## Supporting information

S1 File (DOCX)
