# Supplementary material for: Clinical and economic value of sofosbuvir-based regimens in the treatment of chronic hepatitis C in Spain
Source: PLoS One. 2022 Dec 1;17(12):e0278544. doi: 10.1371/journal.pone.0278544 (PMC9714855; doi:10.1371/journal.pone.0278544)
Supplement: S1 File — (DOCX) [file pone.0278544.s001.docx]

**Supporting Information**

**Table 1S. Parameters used in the analysis for simulation of the progress of hepatitis C chronic**

| **Parameters** | **Base case value** | **Minimum value** | **Maximum value** | **Reference** |
| --- | --- | --- | --- | --- |
| **Annual transition probabilities**  **(from … to)** |  |  |  |  |
| F0 to F1 | 0.131 | ----- | ----- | [4,5] |
| F1 to F2 | 0.080 | ----- | ----- | [4,5] |
| F2 to F3 | 0.133 | ----- | ----- | [4,5] |
| F3 to F4 | 0.134 | ----- | ----- | [4,5] |
| F3 to HCC | 0.011 | ----- | ----- | [4,5] |
| RVS F3 to HCC | 0.003 | ----- | ----- | [4,5] |
| F4 to DC | 0.040 | ----- | ----- | [4,5] |
| F4 to HCC | 0.015 | ----- | ----- | [4,5] |
| SVR F4 to DC | 0.003 | ----- | ----- | [4,5] |
| SVR F4 to HCC | 0.006 | ----- | ----- | [4,5] |
| SVR F4 to Regr. HC | 0.055 | ----- | ----- | [4,5] |
| DC to HCC | 0.068 | ----- | ----- | [4,5] |
| DC to LT | 0.023 | ----- | ----- | [4,5] |
| DC to Liver-related death | 0.138 | ----- | ----- | [4,5] |
| HCC to LT | 0.040 | ----- | ----- | [4,5] |
| HCC to Liver-related death | 0.430 | ----- | ----- | [4,5] |
| LT to Post-LT | 1.000 | ----- | ----- | [4,5] |
| LT to Liver-related death | 0.210 | ----- | ----- | [4,5] |
| Post-LT to Liver-related death | 0.057 | ----- | ----- | [4,5] |
| **Annual health state utility values** |  |  |  |  |
| F0 | 0.98 | 0.92 | 1.00 | [4,5] |
| F1 | 0.98 | 0.92 | 1.00 | [4,5] |
| F2 | 0.92 | 0.72 | 1.00 | [4,5] |
| F3 | 0.79 | 0.77 | 0.81 | [4,5] |
| F4 | 0.76 | 0.70 | 0.79 | [4,5] |
| SVR F0 | 1.00 | 0.98 | 1.00 | [4,5] |
| SVR F1 | 1.00 | 0.98 | 1.00 | [4,5] |
| SVR F2 | 0.93 | 0.92 | 1.00 | [4,5] |
| SVR F3 | 0.83 | 0.82 | 0.90 | [4,5] |
| SVR F4 | 0.83 | 0.79 | 0.87 | [4,5] |
| Regr. HC | 0.86 | 0.82 | 0.90 | [4,5] |
| DC | 0.69 | 0.44 | 0.69 | [4,5] |
| HCC | 0.67 | 0.60 | 0.72 | [4,5] |
| LT | 0.50 | 0.40 | 0.69 | [4,5] |
| Post-LT | 0.77 | 0.57 | 0.77 | [4,5] |
| **Annual health state costs** |  |  |  |  |
| F0 | €272 | €218 | €327 | (±20%) *Assumption* |
| F1 | €272 | €218 | €327 | (±20%) *Assumption* |
| F2 | €315 | €252 | €378 | (±20%) *Assumption* |
| F3 | €315 | €252 | €378 | (±20%) *Assumption* |
| F4 | €573 | €458 | €687 | (±20%) *Assumption* |
| SVR F0 | €116 | €93 | €139 | (±20%) *Assumption* |
| SVR F1 | €116 | €93 | €139 | (±20%) *Assumption* |
| SVR F2 | €116 | €93 | €139 | (±20%) *Assumption* |
| SVR F3 | €116 | €93 | €139 | (±20%) *Assumption* |
| SVR F3 (subsequent years)* | €116 | €93 | €139 | (±20%) *Assumption* |
| SVR F4 | €166 | €133 | €200 | (±20%) *Assumption* |
| SVR F4 (subsequent years)* | €166 | €133 | €200 | (±20%) *Assumption* |
| Regr. HC | €116 | €93 | €139 | (±20%) *Assumption* |
| DC | €2,332 | €1,866 | €2,799 | (±20%) *Assumption* |
| HCC | €8,884 | €7,107 | €10,661 | (±20%) *Assumption* |
| LT | €125,294 | €100,235 | €150,353 | (±20%) *Assumption* |
| Post-LT | €36,623 | €29,298 | €43,948 | (±20%) *Assumption* |
| Post-LT (subsequent years) | €18,331 | €14,649 | €21,974 | (±20%) *Assumption* |

DC: Decompensated Cirrhosis; HCC: Hepatocellular carcinoma; LT: Liver transplant; Post-LT: Post-Liver transplant; Regr. HC: Regression of hepatic cirrhosis; SVR: Sustained Virologic Response.

*The cost of the SVR at F0, F1, F2 and Regr. HC in subsequent years zero euros was assumed**.**

**Table 2S. Results of the deterministic sensitivity analysis**

| **Sensitivity analysis** | **Minimum value** | | **Maximum value** | |
| --- | --- | --- | --- | --- |
|  | **QALYs** | **Total Costs** | **QALYs** | **Total Costs** |
| *Caso Base* | *310765* | *-274M€* | *310,765* | *-274M€* |
| *Univariate* |  |  |  |  |
| DSA1 | 306,207 | -258M€ | ----- | ----- |
| DSA2 | 322,331 | -315M€ | 283,008 | -177M€ |
| DSA3 | 310,765 | -524M€ | 310,765 | -24M€ |
| DSA4 | 310,765 | -175M€ | 310,765 | -374M€ |
| DSA5 | 310,765 | -263M€ | 310,765 | -285M€ |
| DSA6 | 694,856 | -1,273M€ | 195,995 | 59M€ |
| *Multivariate* |  |  |  |  |
| DSA7 | 367,395 | -274M€ | 310,723 | -274M€ |
| DSA8 | 310,765 | -54M€ | 310,765 | -494M€ |
